# Relationship between imaging parameters and distortion in magnetic resonance images for gamma knife treatment planning

**DOI:** 10.1002/acm2.14205

**Published:** 2023-11-17

**Authors:** Norikazu Koori, Hiroki Kamekawa, Nanami Mukawa, Hiraku Fuse, Shin Miyakawa, Kenji Yasue, Masato Takahashi, Masanori Yamada, Atsushi Henmi, Toshifumi Kusumoto, Kazuma Kurata

**Affiliations:** ^1^ School of Health Sciences Ibaraki Prefectural University of Health Sciences Ami Ibaraki Japan; ^2^ Division of Health Sciences Kanazawa University Graduate School of Medical Sciences Kanazawa Ishikawa Japan; ^3^ Department of Radiology Komaki City Hospital Komaki Aichi Japan

**Keywords:** distortion, gamma knife, G‐frame, planning of radiation treatment

## Abstract

In magnetic resonance imaging (MRI), it is necessary to reduce image distortion as much as possible because it suppresses the increase in the planning target volume. This study investigated the relationship between imaging parameters and image distortion when using G‐frames. The images were obtained using a 1.5‐T MRI system with a 09‐101 Pro‐MRI phantom. Image distortion was measured by changing the RF pulse mode, gradient mode, asymmetric echo, and bandwidth (BW). The image distortion was increased in the high RF mode than in the Normal mode. The image distortion increased in the following order: Whisper ≦ Normal < Fast in the different gradient modes. The image distortion increased in the following order: Without ≦ Weak < Strong in the different asymmetric echo modes. The image distortion increased in the following order: 300 Hz/pixel > 670 Hz/pixel ≧ REF (150 Hz/pixel) in the different Bw. The relationship between parameters and image distortion was clarified in this study when G‐frames were used for gamma knife therapy. There is had relationship between the parameters causing variation in the gradient magnetic field and image distortion. Therefore, these parameters should be adjusted to minimize distortion.

## 1 INTRODUCTION

The gamma knife, a stereotactic irradiation (STI) device, is used to treat metastatic brain tumors, auditory nerve tumors, and cerebral artery malformations.^1^, ^2^, ^3^ Computed tomography (CT) and magnetic resonance imaging (MRI) are performed to determine the irradiated area for STI treatment. Generally, the range of the STI was determined by superimposing two images that were obtained by CT and MRI. Although MRI provides excellent contrast between the tumor and normal tissue, the use of metal frames, which is required for gamma knife treatment, causes geometric distortions in the images because of the inhomogeneity of the static magnetic field and the nonlinearity of the gradient magnetic field.^4^, ^5^ Using a special head frame with markers (G‐frame) in the gamma knife system enables the suppression of patient movement and fusion between CT and MRI images, which leads to improved irradiation accuracy. The irradiation accuracy using this G‐frame is reported to be <1 mm.^6^ In gamma knife therapy, it is generally important to lower the dose to the organs involved within 5 mm of the tumor edge to keep them within the tolerable dose range. However, using G‐frames causes greater image distortion than using G‐frameless images,^7^ which it impossible to determine the dose to the tumor and risk organs. When using a 1.5‐T MRI system, MR images used for treatment planning can be distorted by up to 1.9 mm.^8^

Therefore, an appropriate planning target volume (PTV) margin that considers distortion should be determined. Also, it is necessary to determine the parameters of image acquisition that minimize image distortion as much as possible. This large image distortion requires a large PTV and increases the possibility of injury to the normal tissues. Therefore, it is necessary to reduce image distortion as much as possible because it suppresses the increase in PTV. Image distortion can be reduced by adjusting the imaging conditions.^9^ Thus, this study aimed to investigate the relationship between these parameters and image distortion when using G‐frames.

## 2 METHODS

### 2.1 Equipment and imaging conditions

**FIGURE 1:**
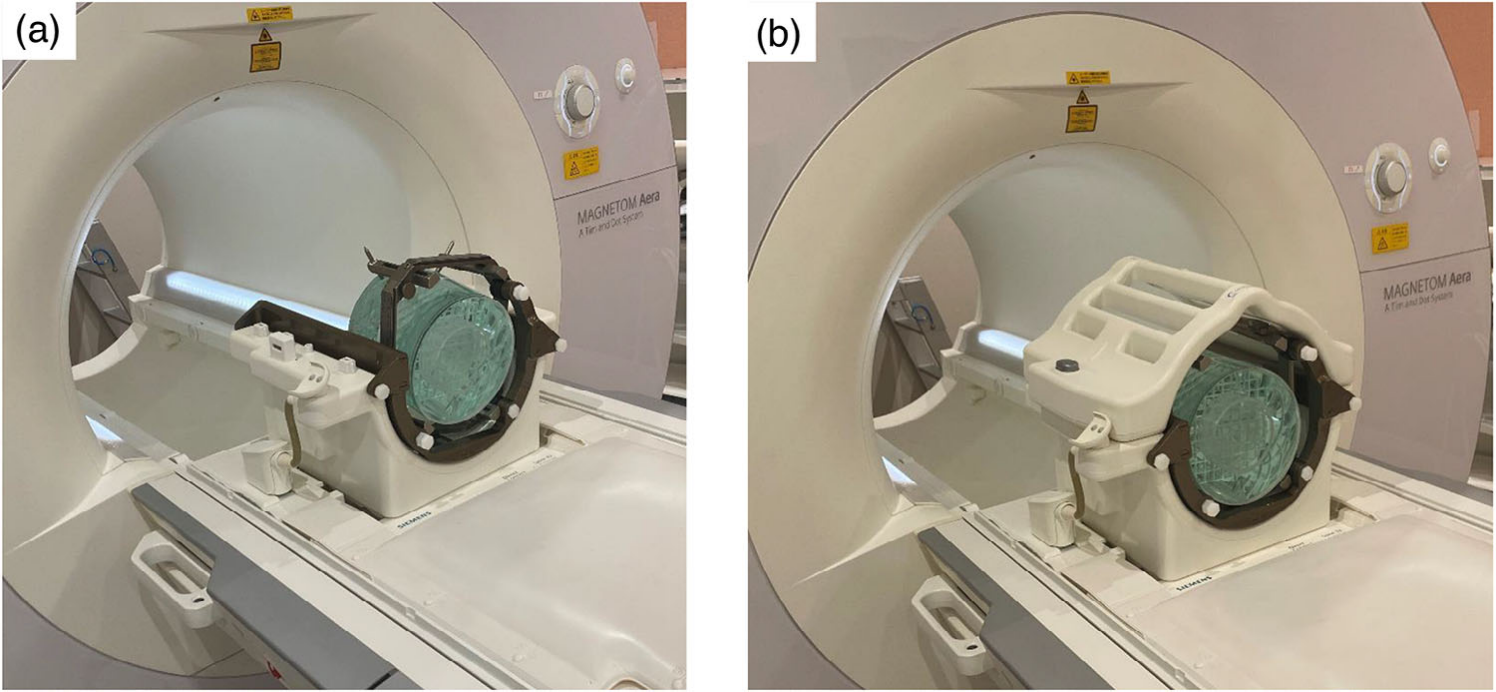
G‐frame phantom placement method. (a) The phantom was fitted with a fixture for gamma‐knife planning. (b) The receiving coil for imaging was attached to it.

The phantom was an American College of Radiology‐compliant 09‐101 Pro‐MRI phantom (Pro‐Project, Okszów, Poland).^10^ The phantom had a nominal value of 173 mm in the axial section and a height of 130 mm. The phantom contained a 10‐mmol/L nickel chloride solution and a 75‐mmol/L sodium chloride solution. A frame used for the gamma knife was attached around this phantom. The phantom was fixed so that the bottom edge of the gamma knife frame and the bottom edge of the phantom were aligned. It was left in an MRI room at 25°C for 24 h before imaging (T_1_, 498.9 ms; T_2_, 137.6 ms). Phantom images are shown in Figure 1.

The phantom was aligned and pictured using LSP‐GNTCNM (TAKENAKA OPTONIC CO., LTD., Kyoto, Japan), an external laser side‐pointer system for treatment planning, to ensure that the phantom was not tilted. Images were acquired 10 times for each imaging condition. The placement of the phantom images is shown in Figure 1(a) and (b). The center of the magnetic field was set at the slice position of axial slice 2.

The imaging conditions are shown in Table 1. The images acquired under these conditions were used as reference images (REF).

**TABLE 1:** Parameter changes for each imaging condition.

Used MRI machine	1.5T MRI (Aera 1.5T, Siemens Healthcare, Erlangen, Germany)							
Used coil	head volume coil							
sequence	Three‐dimensional (3D) volumetric interpolated breath‐hold examination (VIBE)							
Method No.		Method 2‐5	Method 2‐6		Method 2‐7		Method 2‐8	
Method Name	Reference images (REF)	Effects of different radiofrequency pulse modes (RF pulse mode)	Effect of different gradient modes		Effect of asymmetric echo mode		Effects of different bandwidths	
Field of view: FOV (mm)			230					
Slice thicness (mm)			0.8					
Flip angle: FA (°)			10					
Matrix			288×288					
number of signal averages: NSA			1					
The number of slice			208					
3D‐distortion correction (DC)^*^			ON					
Imaging section			Axial					
Repetition time: TR (msec)	8.9	8.9	8.9	9.5	10.4	7.7	5.9	5.4
Echo time: TE (msec)	3.4	3.4	3.4	3.7	4.9	2.3	2	1.5
Radiofrequency pulse mode (RF mode)	Nomal	Fast	Nomal	Nomal	Nomal	Nomal	Nomal	Nomal
Gradient mode	Nomal	Nomal	Fast	Whisper	Nomal	Nomal	Nomal	Nomal
Asymmetric echo mode	Weak	Weak	Weak	Weak	OFF	Strong	Weak	Weak
Band width: BW (Hz/pixel)	150	150	150	150	150	150	300	670
Aquisition time (sec)	655	655	655	703	768	570	439	397

* A distortion correction algorithm provided by the MRI vendor.

### 2.2 Method of calculating signal‐to‐noise ratio (SNR)

**FIGURE 2:**
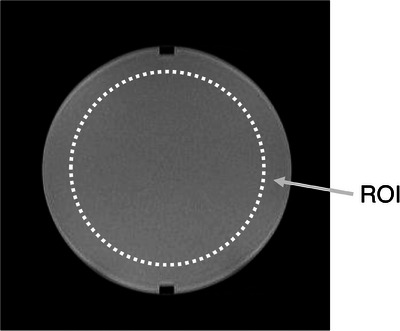
Setting the region of interest method in signal‐to‐noise ratio measurement.

The region of interest (ROIs) size was set at 80% of the diameter of the acquired phantom images, the signal was measured using Image J software (National Institutes of Health, Bethesda, MD, USA),^11^ and the standard deviation was determined using the subtraction method.^12^ The SNR was calculated using Equation (1). Examples of ROIs are shown in Figure 2. The SNR _REF ratio_, the ratio of the SNR of the REF image to the SNR of each image, was calculated using Equation (2).

(1) $$\text{SNR}=\text{Signal}\times \frac{\sqrt{2}}{\text{SD}}$$

(2) $$\mathrm{SN}R_{\mathrm{REF}\;\mathrm{ratio}}=\frac{\mathrm{SN}R_{\mathrm{Actual}}}{\mathrm{SN}R_{\mathrm{REF}}}\;\times 100\left[\%\right]$$

Signal: signal strength, SD: standard deviation, SNR _REF ratio_: ratio of SNR of REF image and SNR of each imaged image, SNR _REF_: SNR of REF image, SNR _Actual_: SNR in each imaged image.

### 2.3 Distortion calculation method

**FIGURE 3:**
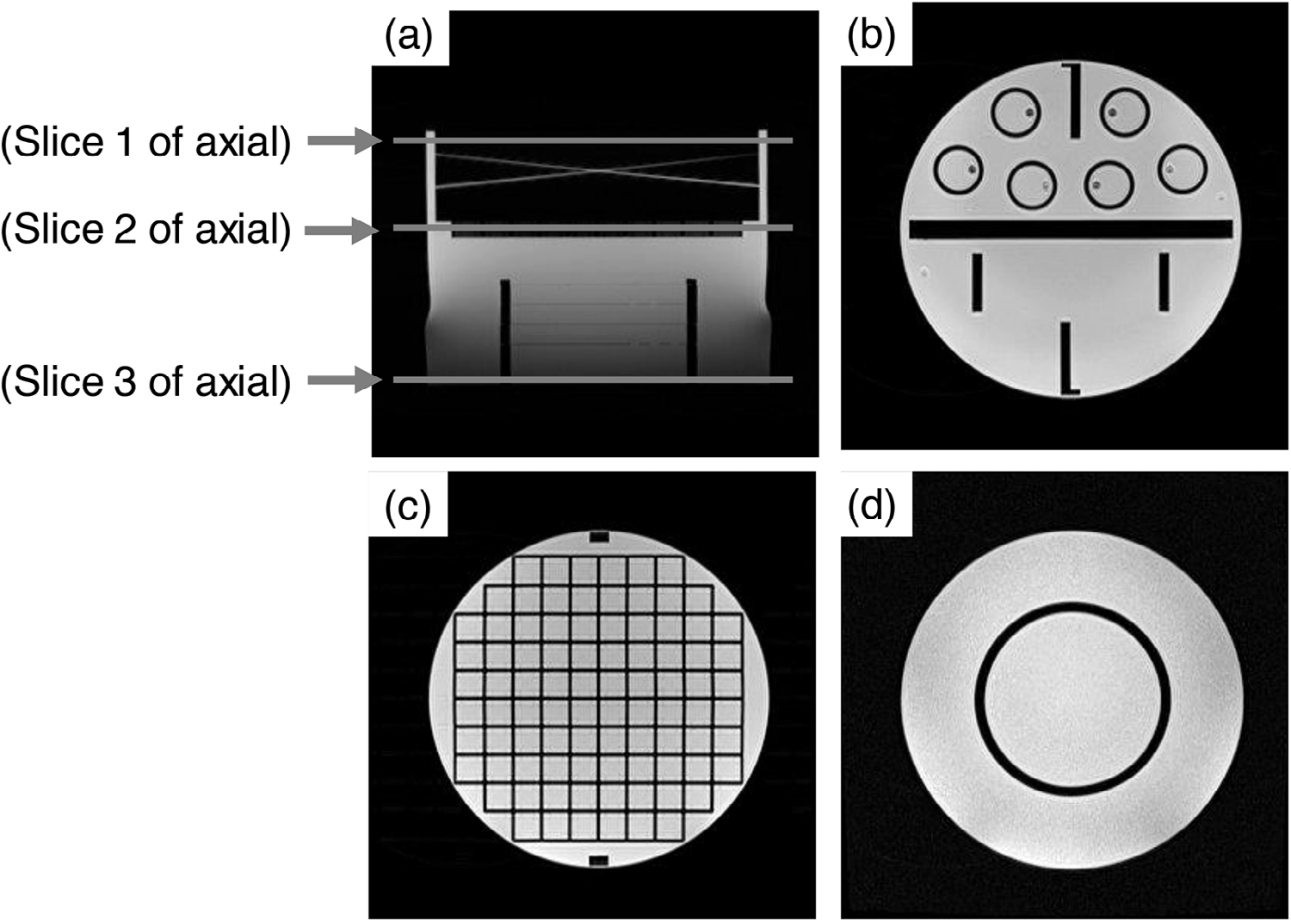
Cross‐section of each axial image. (a) Location of each axial image; (b) Axial image of slice 1 (axial slice 1); (c) Axial image of slice 2 (axial slice 2); and (d) Axial image of slice 3 (axial slice 3).

**FIGURE 4:**
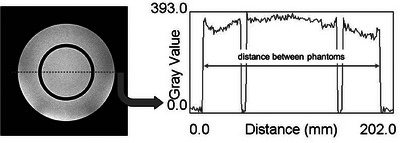
Axial images and their plot profile position. (a) Axial image of slice 1 (axial slice 1); (b) Axial image of slice 2 (axial slice 2); (c) Axial image of slice 3 (axial slice 3).

**FIGURE 5:**
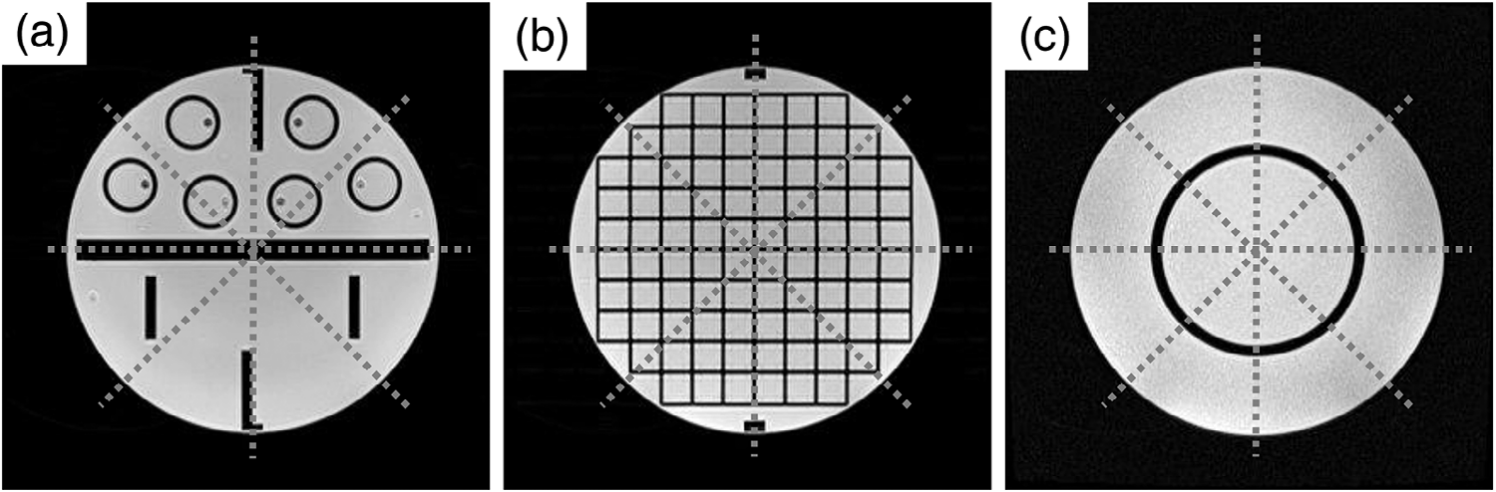
Axial images and plot profile position. (a) Axial image of slice 1 (axial slice 1 of axial); (b) Axial image of slice 2 (axial slice 2); (c) Axial image of slice 3 (axial slice 3).

Image J software was used to select three slices of axial cross‐sectional images obtained via phantom imaging. A coronal image at the location of selecting the three slices of axial cross‐sectional images is shown in Figure 3(a). A cross‐sectional image 2.4 mm foot side from the top of the phantom is shown in Figure 3(b) (axial slice 1), a cross‐sectional section for strain measurement 55 mm foot side from the top of the phantom is shown in Figure 3 (c) (axial slice 2), and a cross‐sectional section 125.6 mm foot side from the top of the phantom is shown in Figure 3(c) (axial slice 3). For the diameter measurement, a profile curve was created using the plot profile function. The distance between phantoms was calculated as the distance between pixels with values greater than half of the average signal intensity obtained in Method 2‐2 (Figure 4). For each slice of the axial image, the phantom diameters were measured at four locations, the distances between the phantoms were calculated at three locations (Figure 5(a)–(c)). Using the diameters and nominal values obtained for the four locations, we calculated the geometric distortion, mean diameter error, and geometric distortion with Equations (3) and (4) for each cross‐section, and the average values for all axial cross‐sections were calculated.

(3) $$\mathrm{MeanDiametererror}\left[\mathrm{mm}\right]=\left|L_{a}-L_{m}\right|$$

(4) $$\mathrm{Geometoricdistortion}\left[\%\right]=\frac{\left|L_{a}-L_{m}\right|}{L_{a}}\times 100$$

where *L_m_* is the measured value of the phantom, and *L_a_* is the actual distance between phantoms (axial image: nominal value of 173 mm). With the above equations, the total axial was the average amount of distortion in the three axial cross‐sections.

Based on the basic parameters of Method 2‐1, the image was obtained by changing the parameters. The mean diameter error and geometric distortion were obtained from images acquired using the distortion calculation method.

### 2.4 Effects of different radiofrequency pulse modes (RF pulse mode)

The images were acquired by changing the RF pulse mode, and image distortion was compared.

### 2.5 Effect of different gradient modes

The images were acquired by changing the gradient mode, and image distortion was compared.

### 2.6 Effect of asymmetric echo mode

The images were acquired by changing the asymmetric echo mode, and image distortion was compared.

### 2.7 Effects of different bandwidths

The images were acquired by changing Bw, and image distortion was compared.

### 2.8 Statistical analysis

Within‐group comparisons were made using Friedman and Wilcoxon signed‐rank tests with Bonferroni correction. A *P*‐value of <0.05 was considered statistically significant. The alpha level was adjusted using Bonferroni correction, if necessary, with the level of significance lowered to 0.05/3 = 0.017 (for paired comparisons among the three groups). R software (version 3.4.1, R Foundation, Vienna, Austria) was used for statistical analysis.

## 3 RESULTS

### 3.1 Effects of different radiofrequency pulse modes

**FIGURE 6:**
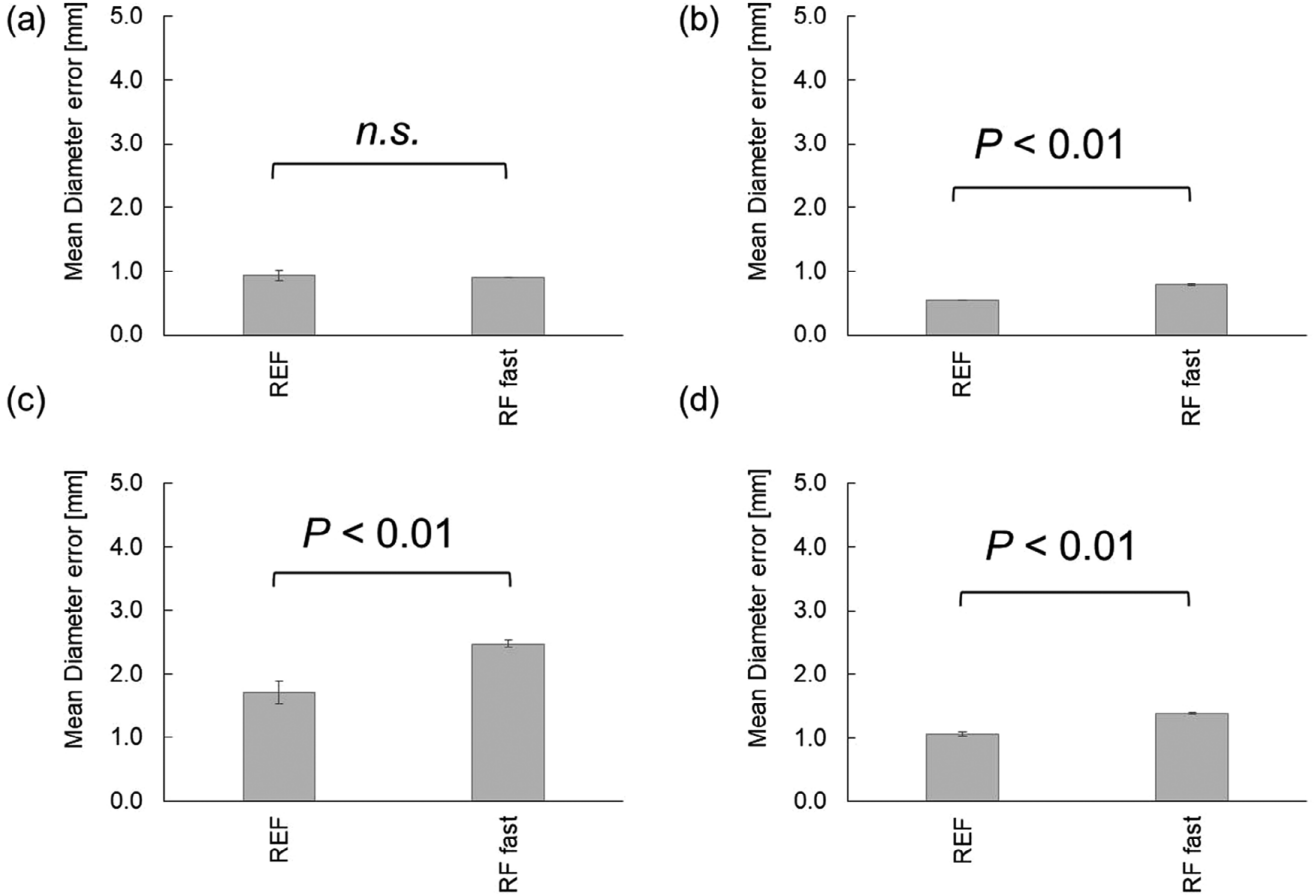
Difference in mean diameter error and geometric distortion between RF pulse modes. (a) at the axial slice 1; (b) at the axial slice 2; (c) at the axial slice 3; (d) average values of the mean diameter error and geometric distortion for all axial slices; (e) in‐plane resolution measurement. REF; with RF pulse mode (Normal), RF fast; RF pulse mode (Fast).

The results are shown in Figure 6(a)–(d). The mean diameter error and geometric distortion at the cross‐section of axial slice 1 were 0.94 ± 0.08 mm (0.54 ± 0.05%) and 0.90 ± 0.00 mm (0.52 ± 0.00%) with REF [RF pulse mode (Normal)] and RF pulse mode (Fast), respectively. No significant differences were found between the two groups.

The mean diameter error and geometric distortion for the cross‐section of axial slice 2 were 0.55 ± 0.00 mm (0.32 ± 0.00%) and 0.79 ± 0.02 mm (0.46 ± 0.01%) with REF [RF pulse mode (Normal)] and RF pulse mode (Fast), respectively. Significant differences were found between the two groups (*P* < 0.01).

The mean diameter error and geometric distortion for the cross‐section of axial slice 3 were 1.71 ± 0.18 mm (0.99 ± 0.10%) and 2.48 ± 0.06 mm (1.43 ± 0.03%) with REF [RF pulse mode (Normal)] and RF pulse mode (Fast), respectively. Significant differences were found between the two groups (*P* < 0.01).

The average values of the mean diameter error and geometric distortion for all axial cross‐sections were 1.06 ± 0.03 mm (0.62 ± 0.02%) and 1.39 ± 0.02 mm (0.80 ± 0.01%) with REF [RF pulse mode (Normal)] and RF pulse mode (Fast), respectively. Significant differences were found between both groups (*P* < 0.01). SNR was 156.1 ± 4.4 and 145.2 ± 3.0 with REF [RF pulse mode (Normal)] and RF pulse mode (Fast), respectively.

### 3.2 Effect of different gradient modes

**FIGURE 7:**
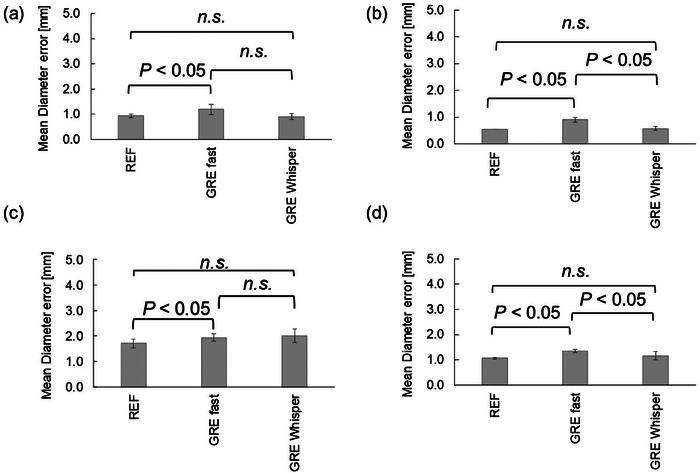
The difference in mean diameter error and geometric distortion between gradient modes. (a) at the axial slice 1; (b) at the axial slice 2; (c) at the axial slice 3; (d) average values of the mean diameter error and geometric distortion for all axial slices; (e) in‐plane resolution measurement. REF; with REF [gradient mode (Normal)], GRE fast; with gradient mode (Fast), GRE Whisper; with gradient mode (Whisper).

The results are shown in Figure 7(a)–(d). The mean diameter error and geometric distortion at the cross‐section of axial slice 1 were 0.91 ± 0.12 mm (0.52 ± 0.07%), 0.94 ± 0.08 mm (0.54 ± 0.05%), and 1.20 ± 0.20 mm (0.69 ± 0.12%) with gradient mode (Whisper), REF [gradient mode (Normal)] and gradient mode (Fast), respectively. Significant differences were found between the REF [Gradient mode (Normal)] and gradient mode (Fast) groups (*P* < 0.05). No significant differences were found between REF [gradient mode (Normal)] and gradient mode (Whisper) groups, or between gradient mode (Fast) and gradient mode (Whisper) groups.

The mean diameter error and geometric distortion for the cross‐section of axial slice 2 were 0.57 ± 0.08 mm (0.33 ± 0.04%), 0.55 ± 0.00 mm (0.32 ± 0.00%), and 0.90 ± 0.09 mm (0.52 ± 0.05%) with gradient mode (Whisper), REF [gradient mode (Normal)] and gradient mode (Fast), respectively. Significant differences were found between the REF [Gradient mode (Normal)] and gradient mode (Fast) groups (*P* < 0.05). No significant differences were found between REF [gradient mode (Normal)] and gradient mode (Whisper) groups, or between gradient mode (Fast) and gradient mode (Whisper) groups.

The mean diameter error and geometric distortion for the cross‐section of axial slice 3 were 2.01 ± 0.27 mm (1.16 ± 0.15%), 1.71 ± 0.18 mm (0.99 ± 0.10%), and 1.94 ± 0.15 mm (1.12 ± 0.09%) with gradient mode (Whisper), REF [gradient mode (Normal)] and gradient mode (Fast), respectively. Significant differences were found between REF [gradient mode (Normal)] and gradient mode (Fast) groups, as well as between gradient mode (Fast) and gradient mode (Whisper) groups (*P* < 0.05). No significant differences were found between REF [Gradient mode (Normal)] and gradient mode (Whisper) groups. The SNR was 150.2 ± 4.6, 156.1 ± 4.4, and 141.0 ± 0.8 with gradient mode (Whisper), REF [gradient mode (Normal)], and gradient mode (Fast), respectively.

The average values of the mean diameter error and geometric distortion for all axial cross‐sections were 1.16 ± 0.15 mm (0.67 ± 0.09%), 1.06 ± 0.03 mm (0.62 ± 0.02%), and 1.35 ± 0.06 mm (0.78 ± 0.03%) with gradient mode (Whisper), REF [gradient mode (Normal)] and gradient mode (Fast), respectively. Significant differences were found between REF [gradient mode (Normal)] and gradient mode (Fast) groups, as well as between gradient mode (Fast) and gradient mode (Whisper) groups (*P* < 0.05). No significant differences were found between REF [Gradient mode (Normal)] and gradient mode (Whisper) groups. The SNR was 150.2 ± 4.6, 156.1 ± 4.4, and 141.0 ± 0.8 with gradient mode (Whisper), REF [gradient mode (Normal)], and gradient mode (Fast), respectively.

### 3.3 Effects of the asymmetric echo mode

**FIGURE 8:**
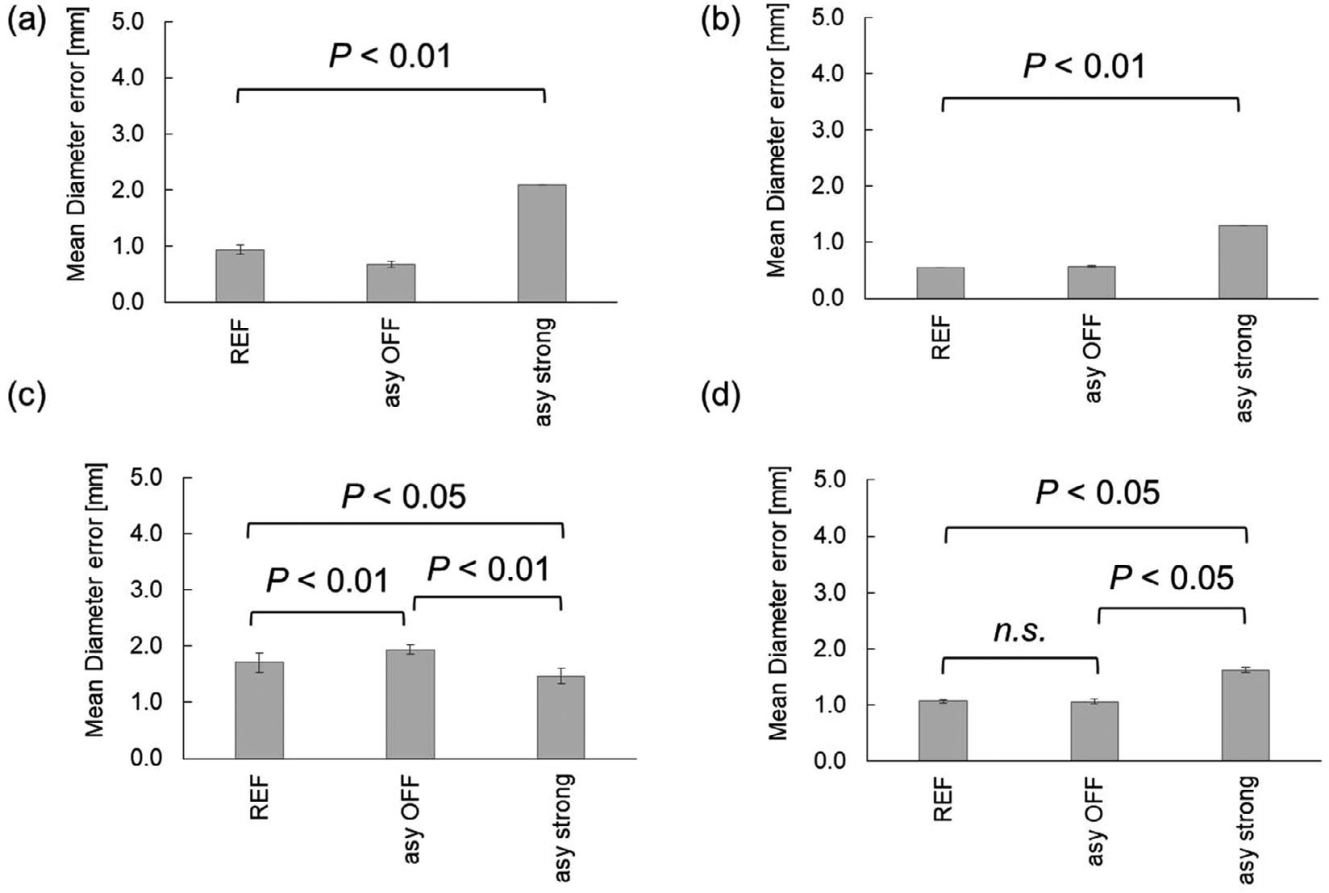
The difference in mean diameter error and geometric distortion between asymmetric echo modes. (a) at the axial slice 1; (b) at the axial slice 2; (c) at the axial slice 3; (d) average values of the mean diameter error and geometric distortion for all axial slices; (e) in‐plane resolution measurement. REF; with REF [asymmetric echo (Weak)], asy OFF; without asymmetric echo, asy Strong; with asymmetric echo (Strong).

The results are shown in Figure 8(a)–(d). The mean diameter error and geometric distortion at the cross‐section of axial slice 1 were 0.68 ± 0.06 mm (0.39 ± 0.03%), 0.94 ± 0.08 mm (0.54 ± 0.05%), and 2.10 ± 0.00 mm (1.21 ± 0.00%) without asymmetric echo, with REF [asymmetric echo (Weak)], and asymmetric echo (Strong), respectively. Significant differences were found among all groups (*P* < 0.01).

The mean diameter error and geometric distortion for the cross‐section of axial slice 2 were 0.57 ± 0.02 mm (0.33 ± 0.01%), 0.55 ± 0.00 mm (0.32 ± 0.00%), and 1.30 ± 0.00 mm (0.75 ± 0.00%) without asymmetric echo, with REF [asymmetric echo (Weak)], and asymmetric echo (Strong), respectively. Significant differences were found among all groups (*P* < 0.01).

The mean diameter error and geometric distortion for the cross‐section of axial slice 3 were 1.94 ± 0.08 mm (1.12 ± 0.05%), 1.71 ± 0.18 mm (0.99 ± 0.10%), and 1.47 ± 0.13 mm (0.85 ± 0.08%) without asymmetric echo, with REF [asymmetric echo (Weak)], and asymmetric echo (Strong), respectively. Significant differences were found between those with REF [asymmetric echo (Weak)] and without asymmetric echo (*P* < 0.05). Significant differences were found between those with REF [asymmetric echo (Strong)] and those without asymmetric echo, as well as between groups with REF [asymmetric echo (Weak)] and those with asymmetric echo (Strong) (*P* < 0.01).

The average values of the mean diameter error and geometric distortion for all axial cross‐sections were 1.06 ± 0.04 mm (0.61 ± 0.02%), 1.06 ± 0.03 mm (0.62 ± 0.02%), and 1.62 ± 0.04 mm (0.94 ± 0.03%) without asymmetric echo, with REF [asymmetric echo (Weak)], and asymmetric echo (Strong), respectively. Significant differences were found between those with REF [asymmetric echo (Weak)] and those with asymmetric echo (Strong), as well as between those without asymmetric echo and those with asymmetric echo (Strong) (*P* < 0.05). No significant differences were found between groups with REF [asymmetric echo (Weak)] and those without asymmetric echo.

The SNR was 134.3 ± 2.4, 156.1 ± 4.4, and 91.9 ± 1.2 without asymmetric echo, with REF [asymmetric echo (Weak)], and with asymmetric echo (Strong), respectively.

### 3.4 Effects of different bandwidths

**FIGURE 9:**
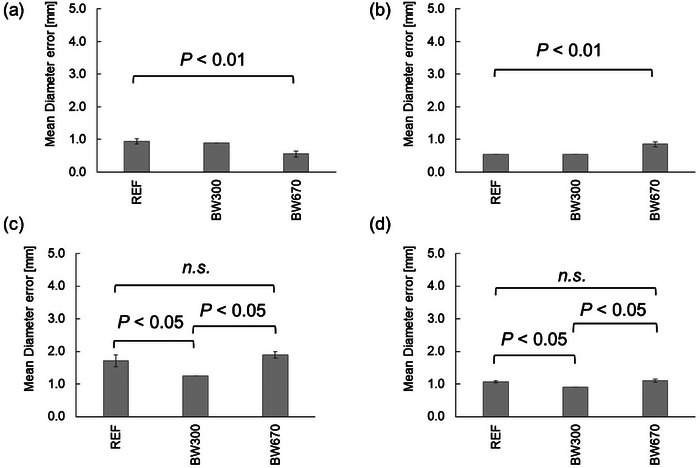
Difference in mean diameter error and geometric distortion between BWs. (a) at the axial slice 1; (b) at the axial slice 2; (c) at the axial slice 3; (d) average values of the mean diameter error and geometric distortion for all axial; (e) in‐plane resolution measurements. REF; with REF [BW = 150 (Hz/pixel)], BW 300; with BW = 300 [Hz/pixel], BW 670; with BW = 670 [Hz/pixel].

The results are shown in Figure 9(a)–(d). The mean diameter error and geometric distortion at the cross‐section of axial slice 1 were 0.94 ± 0.08 mm (0.54 ± 0.05%), 0.90 ± 0.00 mm (0.52 ± 0.00%), and 0.56 ± 0.09 mm (0.32 ± 0.05%) with REF [BW;150 (Hz/pixel)], BW;300 [Hz/pixel], and BW;670 [Hz/pixel], respectively. Significant differences were found between all groups (*P* < 0.01).

The mean diameter error and geometric distortion for the cross‐section of axial slice 2 were 0.55 ± 0.00 mm (0.32 ± 0.00%), 0.55 ± 0.00 mm (0.32 ± 0.00%), and 0.85 ± 0.07 mm (0.49 ± 0.04%) with REF [BW;150 ([Hz/pixel)], BW;300 [Hz/pixel], and BW;670 [Hz/pixel], respectively. Significant differences were found between all groups (*P* < 0.01).

The mean diameter error and geometric distortion for the cross‐section of axial slice 3 were 1.71 ± 0.18 mm (0.99 ± 0.10%), 1.25 ± 0.00 mm (0.72 ± 0.00%), and 1.90 ± 0.10 mm (1.10 ± 0.06%) with REF [BW;150 (Hz/pixel)], BW;300 [Hz/pixel]) and BW;670 [Hz/pixel], respectively. Significant differences were found between groups with REF [BW;150 (Hz/pixel)] and those with BW;670 [Hz/pixel] and between groups with BW;300 [Hz/pixel] and those with BW;670 [Hz/pixel] (*P* < 0.05). No significant differences were found between groups with REF [BW;150 (Hz/pixel)] and those with BW;300 [Hz/pixel].

The average values of the mean diameter error and geometric distortion for all axial cross‐sections were 1.06 ± 0.03 mm (0.62 ± 0.02%), 0.90 ± 0.00 mm (0.52 ± 0.00%), and 1.10 ± 0.05 mm (0.64 ± 0.03%) with REF [BW;150 (Hz/pixel)], BW;300 [Hz/pixel]) and BW;670 [Hz/pixel], respectively. Significant differences were found between groups with REF [BW;150 (Hz/pixel)] and those with BW;670 [Hz/pixel] and between groups with BW;300 [Hz/pixel] and those with BW;670 [Hz/pixel] (*P* < 0.05). No significant differences were found between groups with REF [BW;150 (Hz/pixel)] and those with BW;300 [Hz/pixel]. SNR was 156.1 ± 4.4, 50.4 ± 0.5, and 31.5 ± 0.1 with REF [BW;150 (Hz/pixel)], BW;300 [Hz/pixel]), and BW;670 [Hz/pixel], respectively.

## 4 DISCUSSION

The result 3‐1 shows that the image distortion increased in the RF mode (Fast) than in the Normal mode. When the RF pulse mode is set to Fast, applying a shorter RF pulse irradiation time is possible. Therefore, TR and TE are shortened, and these parameters are used to shorten the aquisition time. When the irradiation time of RF pulses is shortened, the transmission bandwidth widens, resulting in a stronger magnetic field gradient for the same slice thickness.^13^, ^14^ The stronger the magnetic field gradient, the more linearity of the gradient field outside the magnetic field center is decreased. Therefore, the image distortion is considered to have increased. Therefore, Normal is considered to be the best RF pulse mode.

Gradient mode is a parameter related to the slew rate of the gradient magnetic field. The earlier it is set on Fast, the faster the gradient magnetic field can be started up.^14^ Therefore, this parameter is used to shorten TR and TE to reduce the aquisition time. Result 3‐2 shows that image distortion increased in the following order: Whisper ≦ Normal < Fast in the different gradient modes. The fast switching of the gradient magnetic field by setting the gradient mode to Fast caused eddy currents. Therefore, the image distortion is considered to have increased.^14^

The asymmetric echo mode is generally called the partial echo method.^15^ the k‐space is thinned by 25% in the frequency direction with Strong and 12% with Weak using this MRI systems. The theoretical k‐space is filled with the thinned k‐space to shorten the TE compared to the OFF mode. As TE is shortened, TR can also be shortened, enabling a reduction in aquisition time. Also, striped artifacts were observed in the readout direction when the asymmetric echo (readout partial Fourier) was used.^15^ In Result 3−4, the image distortion increased in the following order: OFF ≧ Weak > Strong in the different asymmetric echo modes. The striped artifacts may affect the distortion measurements. Therefore, asymmetric echo mode should not be used.

In Result 3−5, the image distortion increased in the following order: 300 Hz/pixel > 670 Hz/pixel ≧ REF (150 Hz/pixel) in the different Bw. Previous studies have reported that a higher Bw results in less image distortion.^16^, ^17^ However, the increase in BW results in a decrease in SNR. Therefore, reducing image distortion by changing the other parameters evaluated in this study has the advantage of less SNR degradation and is therefore considered easier to use in clinical practice.

MR images are usually distorted because of the nonlinearity of the gradient magnetic field. The further away from the magnetic field center, the more likely image distortion will generally occur.^17^, ^18^, ^19^ However, the DC method corrects the distortion by calculating and predicting the displacement relative to the ideal position and then correcting the position close to the ideal position.^4^ Although it depends on the vendor, image distortion reduction is more effective further away from the magnetic field center when using 3D‐DC.^17^ However, image distortion is significant for equipment that cannot use 3D‐DC. Therefore, setting parameters with low distortion becomes particularly important when using such devices to perform radiotherapy on sites far from the magnetic field center.

In this study, the image distortion was larger in axial slice 1, which corresponds to the head side, under most conditions. This is because axial slice 1 is on the head side compared to axial slices 2 and 3, which correspond to the foot side. Therefore, it is considered that image distortion was more likely to occur because the G‐frame is located in an area where there is more metallic fixed pin for suppression of movement of head. Therefore, it is necessary to understand the distortion tendency depending on the position of the G‐frame when planning treatment.

This study used the G‐frame and fixed pins of the Leksell stereotactic system. However, the amount of image distortion differs depending on the pin and frame materials.^20^ Therefore, it is possible to further reduce image distortion by changing the material of the frame and using the fixed pin. In the Association of Physicists in Medicine Task Group 1 Report No. 100,^21^ the allowable image distortion for MRI for radiotherapy planning was defined as 2.0% (2.0 mm). The parameters considered in this study exceed the acceptable values depending on the aquisition position.

Further, guidelines and statements is mentioned that the accuracy of stereotactic radiotherapies, such as gamma knifes, should be within 1 mm.^22^ Therefore, it is necessary to select imaging conditions that reduce image distortion as much as possible. Consequently, the PTV margin is smaller, which may reduce damage to normal tissues and improve the treatment results.

Therefore, minimizing distortion by combining the parameters considered in this study is possible. Also, it is important to understand the parameters that affect distortion among those that can be set and to set the parameters that cause the least distortion. Therefore, other parameters not chosen in this study should be considered in the future for further distortion reduction.

This study had some limitations. First, a 70‐cm‐bore device was used, but the larger the bore size, the greater the distortion and effect due to the length of the magnet.^23^ Additionally, the further away from the center of the magnetic field, the more likely it is that effects due to differences between the devices will occur. Therefore, distortions may differ between manufacturers and devices. Second, the accuracy of strain measurement depends on the voxel size. The MRI system used in this study had the same spatial resolution in all phases, frequencies, and slice directions. A voxel size of 0.8 mm was the minimum value to image the entire phantom. Therefore, an error of 0.8 mm would occur if only one pixel was misplaced in the pixels to be measured. A higher resolution is required to measure detailed image distortion. Third, the phantom used in this study is not a dedicated phantom for strain measurement. Therefore, the results may be more precise if measurements are performed on a dedicated phantom for strain measurement. Fourth, this study was conducted by a single vendor. Therefore, the results may differ depending on the vendor and the version of the device used. It is necessary to confirm the effect of distortion reduction with each device. However, the general trend of distortion reduction is likely to be the same. Fifth, this study shows that image distortion can be significantly reduced by increasing Bw. However, the Bw of the REF imaging conditions in this study was 150 Hz/pixel. Even if other parameters were varied while Bw of the REF imaging conditions was set at 300 or 670 Hz/pixel, the distortion reduction effect may have been small because an increase in Bw alone can minimize distortion. Therefore, future research is required to clarify the effect of each parameter with Bw set to the maximum acceptable value.

## 5 CONCLUSION

The relationship between parameters and image distortion was clarified in this study when G‐frames used for gamma knife therapy were used. There was a close relationship between the parameters that give variation to the gradient magnetic field and image distortion. Therefore, these parameters should be adjusted to minimize distortion.

## AUTHOR CONTRIBUTIONS

Norikazu Koori: Collected the data, conceived and designed the analysis, wrote the paper. Hiroki Kamekawa: Acquired data, revising it critically for important intellectual content. Nanami Mukawa: Data analysis, revising it critically for important intellectual content. Hiraku Fuse: Revising it critically for important intellectual content. Shin Miyakawa: Revising it critically for important intellectual content. Kenji Yasue: Revising it critically for important intellectual content. Masato Takahashi: Revising it critically for important intellectual content. Masanori Yamada: Revising it critically for important intellectual content. Atsushi Henmi: Revising it critically for important intellectual content. Toshifumi Kusumoto: Revising it critically for important intellectual content. Kazuma Kurata: Revising it critically for important intellectual content.

## CONFLICT OF INTEREST STATEMENT

The authors declare no conflicts of interest.

## Data Availability

Data are available on request from the authors.

## References

[1] Muacevic A , Kreth FW , Horstmann GA , et al. Surgery and radiotherapy compared with gamma knife radiosurgery in the treatment of solitary cerebral metastases of small diameter. J Neurosurg. 1999;91(1):35‐43. 10389878 10.3171/jns.1999.91.1.0035

[2] Kobayashi T , Tanaka T , Kida Y . The early effects of gamma knife on 40 cases of acoustic neurinoma. Acta Neurochir Suppl. 1994;62:93‐97. 7717145 10.1007/978-3-7091-9371-6_19

[3] Kawashima M , Hasegawa H , Shin M , et al. Long‐term outcomes of stereotactic radiosurgery for ruptured arteriovenous malformations. Surg Cereb Stroke. 2022;50:20‐24. [in Japanese].

[4] Wang D , Doddrell DM , Cowin G . A novel phantom and method for comprehensive 3‐dimensional measurement and correction of geometric distortion in magnetic resonance imaging. Magn Reson Imaging. 2004;22(4):529‐542. 15120173 10.1016/j.mri.2004.01.008

[5] Janke A , Zhao H , Cowin GJ , Galloway GJ , Doddrell DM . Use of spherical harmonic deconvolution methods to compensate for nonlinear gradient effects on MRI images. Magn Reson Med. 2004;52(1):115‐122. 15236374 10.1002/mrm.20122

[6] Claps L , Mathew D , Dusenbery K , Reynolds M , Watanabe Y . Utilization of CBCT to improve the delivery accuracy of gamma Knife radiosurgery with G‐frame. J Appl Clin Med Phys. 2021;22(8):120‐128. 10.1002/acm2.13332 PMC8364265 34196098

[7] Pappas EP , Seimenis I , Moutsatsos A , Georgiou E , Nomikos P , Karaiskos P . Characterization of system‐related geometric distortions in MR images employed in gamma Knife radiosurgery applications. Phys Med Biol. 2016;61(19):6993‐7011. 27648985 10.1088/0031-9155/61/19/6993

[8] Yu C , Apuzzo ML , Zee CS , Petrovich Z . A phantom study of the geometric accuracy of computed tomographic and magnetic resonance imaging stereotactic localization with the Leksell stereotactic system. Neurosurgery. 2001;48(5):1092‐1099. 11334276 10.1097/00006123-200105000-00025

[9] Retif P , Djibo Sidikou A , Mathis C , et al. Evaluation of the ability of the brainlab elements cranial distortion correction algorithm to correct clinically relevant MRI distortions for cranial SRT. Strahlenther Onkol. 2022;198(10):907‐918. 35980455 10.1007/s00066-022-01988-1

[10] American College of Radiology . Large phantom Slice 5 grid guidance. Accessed September 10, 2022. https://www.acraccreditation.org/‐/media/ACRAccreditation/Documents/MRI/Slice‐5‐Guidance.pdf

[11] Abramoff MD , Magalhaes PJ , Ram SJ . Image processing with Image. J Biophotonics Int. 2004;11(7):36‐42.

[12] National Electrical Manufacturers Association: Determination of Signal‐to‐Noise Ratio (SNR) in Diagnostic Magnetic Resonance Imaging. NEMA Standards Publication MS 1‐2008 (R2014).

[13] Joachim G , Bandwidth in MRI? MAGNETOM Flash, 2013. Accessed June 23, 2023. https://marketing.webassets.siemens‐healthineers.com/1800000000456951/fd0ec723f13f/bandwith_in_mri_1800000000456951.pdf

[14] Araki T , ketteiban MRIkannzennkaisetsu. 2014. Gakken Medical Shujunsha Co., Ltd., Japan, Tokyo. [in Japanese].

[15] Yoshimura Y , Suzuki D , Miyahara K . Evaluation of image quality of readout segmented EPI with readout partial Fourier technique. Nihon Hoshasen Gijutsu Gakkai Zasshi. 2017;73(12):1244‐1251. 29269620 10.6009/jjrt.2017_JSRT_73.12.1244

[16] Walker A , Liney G , Metcalfe P , Holloway L . MRI distortion: considerations for MRI based radiotherapy treatment planning. Australas Phys Eng Sci Med. 2014;37(1):103‐113. 24519001 10.1007/s13246-014-0252-2

[17] Slagowski JM , Ding Y , Aima M , et al. A modular phantom and software to characterize 3D geometric distortion in MRI. Phys Med Biol. 2020;65(19):195008. 32531763 10.1088/1361-6560/ab9c64 PMC7772054

[18] Seibert TM , White NS , Kim GY , et al. Distortion inherent to magnetic resonance imaging can lead to geometric miss in radiosurgery planning. Pract Radiat Oncol. 2016;6(6):e319‐e328. 27523440 10.1016/j.prro.2016.05.008 PMC5099096

[19] Alzahrani M , Broadbent DA , Chuter R , et al. Audit feasibility for geometric distortion in magnetic resonance imaging for radiotherapy. Phys Imaging Radiat Oncol. 2020;15:80‐84. 33163632 10.1016/j.phro.2020.07.004 PMC7607582

[20] Nakazawa H , Mori Y , Yamamuro O , et al. Geometric accuracy of 3D coordinates of the Leksell stereotactic skull frame in 1.5 Tesla‐ and 3.0 Tesla‐magnetic resonance imaging: a comparison of three different fixation screw materials. J Radiat Res. 2014;55(6):1184‐1191. 25034732 10.1093/jrr/rru064 PMC4229929

[21] Edward FJ , Michael JB , Dick JD , et al. Acceptance testing and quality assurance procedures for magnetic resonance imaging facilities. AAPM Report No. 100; 2010.

[22] Japanese Society for Radiation Oncology. The Committee on Quality assurance of JASTRO 2016; 2016. KANEHARA, Co LTD, Japan T. Japanese. Accessed September 10, 2022. [in Japanese]. https://www.jastro.or.jp/medicalpersonnel/guideline/qa_guidline2016.pdf

[23] Yasuda S , Yamakoshi K . Study of the usefulness of the 3D‐distortion correction in MRI. Nihon Hoshasen Gijutsu Gakkai Zasshi. 2016;72(9):746‐756. [in Japanese]. 27647597 10.6009/jjrt.2016_JSRT_72.9.746

